# Nectin-4-targeted immunoSPECT/CT imaging and photothermal therapy of triple-negative breast cancer

**DOI:** 10.1186/s12951-022-01444-3

**Published:** 2022-05-25

**Authors:** Fuqiang Shao, Zhidi Pan, Yu Long, Ziyang Zhu, Kun Wang, Hao Ji, Ke Zhu, Wenyu Song, Yangmeihui Song, Xiangming Song, Yongkang Gai, Qingyao Liu, Chunxia Qin, Dawei Jiang, Jianwei Zhu, Xiaoli Lan

**Affiliations:** 1 Department of Nuclear Medicine, Union Hospital, Tongji Medical College, Huazhong University of Science and Technology, No. 1277 Jiefang Ave, Wuhan, 430022 China; 2 Hubei Province Key Laboratory of Molecular Imaging, Wuhan, 430022 China; 3 Department of Nuclear Medicine, Zigong First People’s Hospital, Zigong Academy of Medical Sciences, Zigong, 643000 China; 4 Engineering Research Center of Cell & Therapeutic Antibody, Ministry of Education, School of Pharmacy, Shanghai Jiao Tong University, 800 Dongchuan Road, Shanghai, 200240 China; 5 Jecho Laboratories, Inc., Frederick, MD 21704 USA; 6 Jecho Biopharmaceuticals Co., Ltd., Tianjin, 300467 China; 7 Key Laboratory of Biological Targeted Therapy , the Ministry of Education , Wuhan, 430022 China

**Keywords:** Triple-negative breast cancer, Indocyanine green, Single photon emission computed tomography, Photothermal therapy, Nectin-4

## Abstract

**Background:**

Triple-negative breast cancer (TNBC) is more prone to distant metastasis and visceral recurrence in comparison to other breast cancer subtypes, and is related to dismal prognosis. Nevertheless, TNBC has an undesirable response to targeted therapies. Therefore, to tackle the huge challenges in the diagnosis and treatment of TNBC, Nectin-4 was selected as a theranostic target because it was recently found to be highly expressed in TNBC. We developed anti-Nectin-4 monoclonal antibody (mAb_Nectin-4_)-based theranostic pair, ^99m^Tc-HYNIC-mAb_Nectin-4_ and mAb_Nectin-4_-ICG. ^99m^Tc-HYNIC-mAb_Nectin-4_ was applied to conduct immuno-single photon emission computed tomography (SPECT) for TNBC diagnosis and classification, and mAb_Nectin-4_-ICG to mediate photothermal therapy (PTT) for relieving TNBC tumor growth.

**Methods:**

Nectin-4 expression levels of breast cancer cells (MDA-MB-468: TNBC cells; and MCF-7, non-TNBC cells) were proved by western blot, flow cytometry, and immunofluorescence imagning. Cell uptake assays, SPECT imaging, and biodistribution were performed to evaluate Nectin-4 targeting of ^99m^Tc-HYNIC-mAb_Nectin-4_. A photothermal agent (PTA) mAb_Nectin-4_-ICG was generated and characterized. In vitro photothermal therapy (PTT) mediated by mAb_Nectin-4_-ICG was conducted under an 808 nm laser. Fluorescence (FL) imaging was performed for mAb_Nectin-4_-ICG mapping in vivo. In vivo PTT treatment effects on TNBC tumors and corresponding systematic toxicity were evaluated.

**Results:**

Nectin-4 is overexpressed in MDA-MB-468 TNBC cells, which could specifically uptake ^99m^Tc-HYNIC-mAb_Nectin-4_ with high targeting in vitro*.* The corresponding immunoSPECT imaging demonstrated exceptional performance in TNBC diagnosis and molecular classification. mAb_Nectin-4_-ICG exhibited favourable biocompatibility, photothermal effects, and Nectin-4 targeting. FL imaging mapped biodistribution of mAb_Nectin-4_-ICG with excellent tumor-targeting and retention in vivo. Moreover, mAb_Nectin-4_-ICG-mediated PTT provided advanced TNBC tumor destruction efficiency with low systematic toxicity.

**Conclusion:**

mAb_Nectin-4_-based radioimmunoimaging provides visualization tools for the stratification and diagnosis for TNBC, and the corresponding mAb_Nectin-4_-mediated PTT shows a powerful anti-tumor effect. Our findings demonstrate that this Nectin-4 targeting strategy offers a simple theranostic platform for TNBC.

**Supplementary Information:**

The online version contains supplementary material available at 10.1186/s12951-022-01444-3.

## Introduction

Breast cancer is the most common malignancy affecting global females, surpassing lung cancer to become the highest-ranking cancer type in 2020 [1]. Breast cancer is a heterogeneous entity, and the subtypes grouping could be basing on the expression status of progesterone receptor (PR), oestrogen receptor (ER), and human epidermal growth factor receptor 2 (HER2) in the tumors [2]. Among them, tumors that do not express all of ER, PR and Her-2 are described as triple-negative breast cancer (TNBC).

Compared to other subtypes, TNBC is more prone to distant metastasis and visceral recurrence and is generally related to an unfavorable prognosis [3–5]. In addition, TNBC affects younger patients more frequently than the other subtypes [6]. Because of the lack of PR, ER, and HER2, endocrine therapy and HER2-targeted therapy cannot be undertaken for TNBC, and the therapeutic strategy is mainly confined to chemotherapy [7, 8]. However, drug resistance is a huge obstacle [9]. The aggressive nature of TNBC and the lack of effective targeted therapy have brought significant challenges to its clinical diagnosis and treatment. Therefore, cell surface proteins that are specifically expressed in TNBC cells but not expressed or downregulated in normal breast tissue will be ideal diagnostic biomarkers and therapeutic targets.

Nectin-4, namely poliomyelitis virus receptor related protein 4, is a transmembrane protein that mediates Ca2^+^-independent cell adhesion [10, 11]. The expression of Nectin-4 is mainly occurred during embryogenesis, which declined in adult life. Nectin-4 is hardly expressed in adult tissues and serum [10]. Overexpression of Nectin-4 is observed in various human tumors, including bladder, pulmonary, pancreatic, gastric, esophageal, and ovarian cancer [12–16]. Recent studies revealed that Nectin-4 is expressed in 62% of TNBC and is associated with poor prognosis [17]. Given the limited expression in normal human tissues and overexpression in TNBC [17, 18], Nectin-4 is an attractive candidate as a novel therapeutic biomarker for TNBC [19]. To identify TNBC patients who can benefit from Nectin-4-related therapy, it is necessary to detect Nectin-4 expression levels in tumors.

Owing to inter- and intra-tumoral heterogeneity and sampling dependence, however, biopsies cannot accurately assess tumor phenotype and involve an invasive examination that limits their application. Radionuclide imaging is a highly sensitive non-invasive visualization tool that can address this challenge [20, 21]. Immuno-single photon emission computed tomography (SPECT) and immuno-positron emission tomography (PET) applies radiolabelled monoclonal antibodies and derivatives as tracers for imaging, and can visualize biomarker distribution and detect expression levels in vivo [22–27].

In recent years, several novel therapeutic strategies of TNBC have emerged, such as antibody–drug conjugate therapy (ADC) [28], immune cell therapy, and photodynamic therapy/photothermal therapy (PDT/PTT) [29–31]. Among them, PTT has been widely demonstrated as a prospective non-invasive therapeutic regimen for cancer. PTT delivers photosensitiser to tumor tissues with high light-to-heat conversion performance and uses near-infrared (NIR) light radiation to generate heat to kill cancer cells and suppress tumor growth [32, 33].

The efficacy of PTT depends on two principal factors. One is the carrier, which accurately delivers the photothermal agent (PTA) to the tumor site. Through antigen–antibody-specific binding, whereby anti Nectin-4 mAb can act as the deliverer. The other is appropriate PTA. Near infrared (NIR) light has good penetration of biological tissues, and NIR-absorbing materials demonstrate great development potential in clinical diagnosis and PTT treatment of disease [32]. Indocyanine green (ICG) is Food and Drug Administration (FDA)-approved clinically used dye, which has an excitation range of 700–900 nm, with advantages of low toxicity and rapid metabolism [34]. In 1997, Chen et al. [35] first used ICG to perform PTT in preclinical tumor models under 805 nm laser and verified its effectiveness. Li et al. [36] reported the clinical translation of the ICG-mediated PTT strategy in local tumor ablation of breast cancer. The results showed that the objective remission rate of target lesions was 62.5% with low toxicity and few side effects [36].

**Scheme 1:**
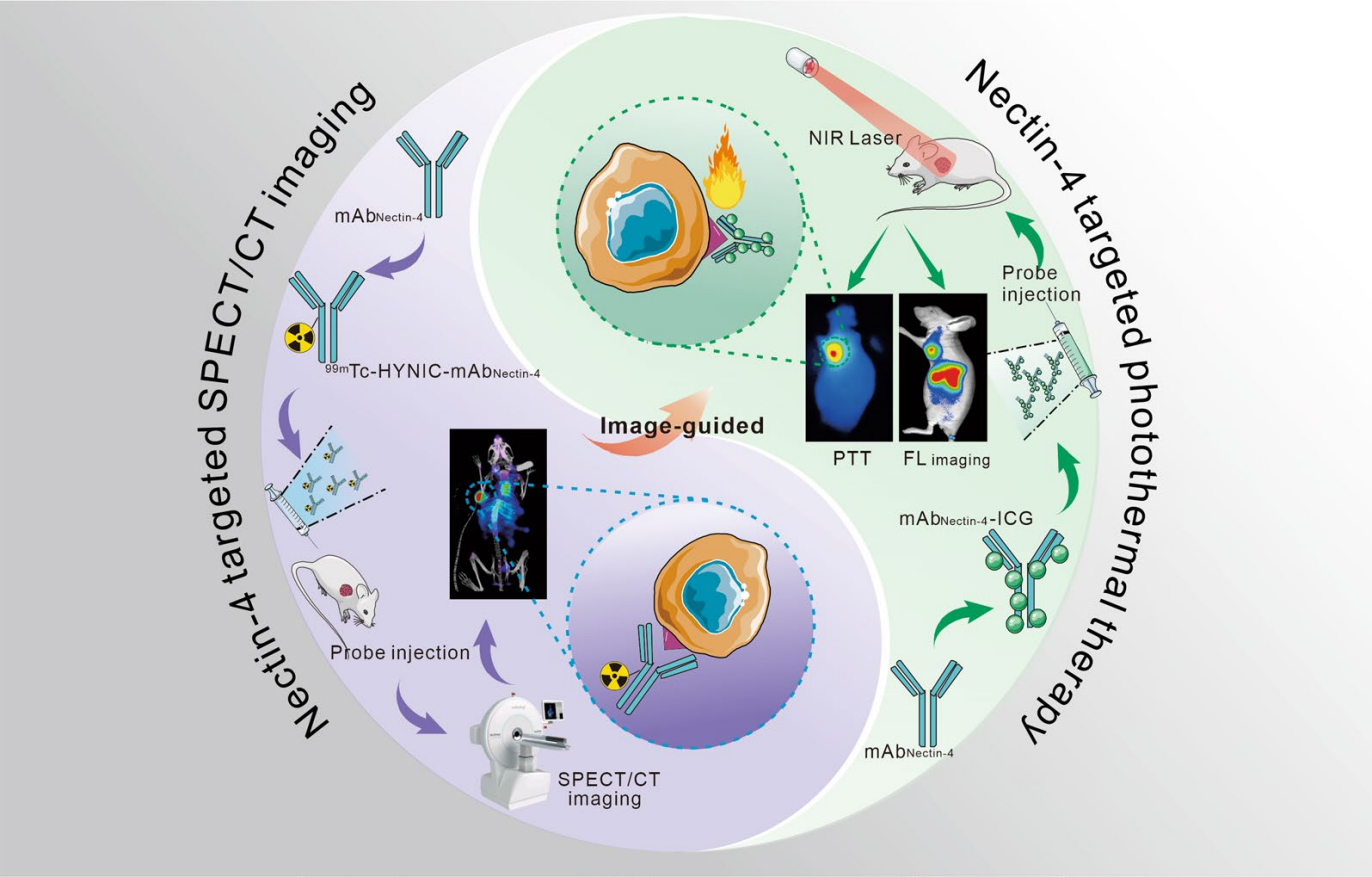
Illustration of the mAb_Nectin-4_ mediated SPECT/CT and corresponding image-guided PTT for TNBC

In this study, using Nectin-4 as a biomarker, we employed Nectin-4-targeted mAb as a carrier to mediate immuno-SPECT imaging and PTT for TNBC (Scheme 1).

## Materials and methods

### Antibody preparation

Genes of anti Nectin-4 antibody were generated (General Biosystems, Anhui, China), and subcloned into a pCDNA 3.4 vector (Invitrogen). HEK 293F cells were transiently transfected with plasmids loading antibody genes and cultured. The supernatant containing anti Nectin-4 antibody was collected for antibody extraction and purification as previously described [37].

### Cell culture

Cell lines (TNBC, MDA-MB-468; non-TNBC, MCF-7) were provided by the Shanghai Type Culture Collection of the Chinese Academy of Sciences. MDA-MB-468 cells were cultivated with modified L-15 medium (Gibco, USA) containing 10% fetal bovine serum (FBS) (Gibco, USA) and 1% penicillin–streptomycin at 37 °C under a humidified atmosphere with 0% CO_2_. MCF-7 cells were cultivated with DMEM medium (10% FBS + 1% penicillin–streptomycin; 37 °C, 5% CO_2_).

### Western blot

When the growth density of MDA-MB-468 and MCF-7 cells reached about 80%, cells were dispersed and collected for protein extraction. The total protein concentration was measured and the protein (20 µg each lane) was added to 10% sodium dodecyl sulfate polyacrylamide gel electrophoresis (SDS-PAGE). After separation, trarsmembran was performed using polyvinylidene fluoride (PVDF) film. The film was incubated with the primary antibody (Nectin-4 antibody; Abcam, ab192033; 4 °C, overnight) and secondary antibody (goat anti-rabbit IgG, 1:10,000; 4 °C, 2 h) orderly. The films were visualized on a Visionwork system. Glyceraldehyde 3‑phosphate dehydrogenase (GAPDH) was applied as internal reference.

### Flow cytometry

The cells were collected and suspended in phosphate buffer saline (PBS) (concentration: 1 × 10^6^ cells/100 μL). After dispersing, cells were incubated with Cy5 labeled mAb_Nectin-4_ on ice for 30 min. For blocking assay, pre-treat the MDA-MB-468 cells using excess unlabeled mAb_Nectin-4_ 1 h prior to mAb_Nectin-4_-Cy5 incubation. After flushing with PBS for three times, cell were tested by FACSCalibur flow cytometer (Becton,Dickinson and Company, USA).

### Confocal laser scanning microscopy (CLSM) imaging

Seed the cells in 35 mm plates (density: 1 × 10^4^) for incubation overnight. Replace 1 mL serum-free medium containing Cy5 labeled mAb_Nectin-4_ (20 µg/mL) to each plate and incubation was performed for 1 h. Rinse the Cy5-treated cells with pre-cooling PBS for three times, 200 μL 4% paraformaldehyde solution was added to fix cells. Then DAPI and FITC-Phalloidin was used for nuclear and cytoskeleton staining respectively. Images with different channels (Excitation/Emission of the Cy5, DAPI, and FITC were 650/670 nm, 360/450 nm, and 490/520 nm, respectively) were acquired by fluorescence optics of a confocal microscope (Zeiss, NOL-LSM 710). For blocking studies, MDA-MB-468 cells were incubated with excess mAb_Nectin-4_ for 1 h previously before experiments.

### Radiolabeling of mAb_Nectin-4_ with ^99m^Tc

Nectin-4-specific mAb (mAb_Nectin-4_) was purified by Zeba™ desalting column (7 K, 0.5 mL) first. Fourty µg bifunctional chelator succinimidyl 6-hydraziniumnicotinate hydrochloridem (SHNH), (Solulink, Inc., USA) was added to the purified mAb_Nectin-4_ (150 µg, 1 nmol) and reacted overnight at 4 °C for the synthesis of Hynic-conjugated mAb_Nectin-4_. After filtering with Zeba™ desalting column to remove the excess SHNH, tricine solution (100 μL,100 mg/mL), SnCl2 solution (4 μL, 7 mg/mL) and ^99m^TcO_4_^−^ eluate (500 μL, 1110 MBq; eluted from ^99m^Tc/^99^Mo generator) were added orderly into the Hynic-conjugated mAb_Nectin-4_ solution. Thhe mixture was incubated away from light at 25 °C for 30 min, then purified with PD-10 desalting column (GE, USA). The labeling efficiency (prior to PD-10 purification) and radiochemical purity (post to PD-10 purification) were examined with instant thin layer chromatography (ITLC; mobile phase: PBS).

### Cell uptake assays

Seed the cells were in 24-well plates with a cell concentration of 1.0 × 10^5^ cells/well. After complete attachment, replace 1 mL serum-free medium containing ^99m^Tc-Hynic-mAb_Nectin-4_ (37 kBq) to each well and incubation was performed at 37 °C for multiple time points (1, 2, 3, 4,6, and 8 h). For each time point, rinse the radiotracer-treated cells with 800 μL pre-cooling PBS twice. Collect the rinsed PBS as supernatants. Then 800 µL NaOH (1 N) was added for cell lysis and collected as lysates. Radioactivity of the supernatants and lysates was detected using automatic gamma-counter (PerkinElmer, USA). After attenuation correction, the cell uptake rate is determined as: A_lysate_/(A _supernatant_ + A_lysate_) × 100%. For blocking studies, experimental cells were incubated with excess unradiolabeled mAb_Nectin-4_ for 1 h before experiments.

### Animals and tumor modeling

All experimental animal examinations were conducted following Institutional Animal Care and Use Committee of Huazhong University of Science and Technology-approved protocol. 150 µL cell suspension (containing 1 × 10^7^ MDA-MB-468 or MCF-7 cells, Matrigel and sterile PBS mixed in a ratio of 1:1) was implanted into the right axilla to each mouse (Balb/c-nude, female, aged between 4 and 6 weeks,) for subcutaneous xenograft inoculation. Mice with a tumor volumes of 200–300 mm^3^ were applied for in vivo imaging and that of 100 mm^3^ for PTT.

### SPECT/CT imaging and ex vivo biodistribution

All SPECT/CT images were scanned by microPET/SPECT/CT multimodal imaging system (InliView-3000B, Novel Medical™, Yongxin Medical Equipment Co., Ltd., Beijing, China). Thirty-seven MBq ^99m^Tc-Hynic-mAb_Nectin-4_ (about 10 µg mAb, within 150 µL PBS) was administrated via the tail vein. Maximum intensity projection (MIP) and transaxial images were acquired at 3, 6, 12, 24, and 36 h post injection (p.i.). Non-contrast-enhanced CT (50 keV, 0.5 mA) was performed for the attenuation-correction of SPECT data. Anaesthetization was perfomed with isofurane amid the scanning duration. For the blocking study, MDA-MB-468 xenograft tumor-bearing mice received excess cool mAb_Nectin-4_ (1 mg) 2 h prior to the radiotracer injection. For semi-quantitative analysis, the regions of interest (ROIs) of the tumor and the contralateral normal muscle were delineated on selected transaxial images and the radioactivity count was obtained. The T/M ratio was calculated as count_tumor_/count_muscle_.

The ex vivo biodistribution studies were performed to quantify the ^99m^Tc-Hynic-mAb_Nectin-4_ uptake in relevant organs. After the last time-point of SPECT/CT imaging, sacrifice the mice and collect the chosen organs/tissues (the blood, brain, heart, lung, liver, spleen, kidney, pancreas, stomach, small intestine, large intestine, muscle, bone, and the tumor). Following washing with PBS, weights and radioactivity of each organ were examined. After attenuation correction, the uptake of each tissue was computed and described as %ID/g. For semi-quantitative analysis, the tumor to blood (T/B) and tumor to muscle (T/M) radioactivity ratio were also computed.

### Synthesis and characterization of mAb_Nectin-4_-ICG

ICG-NHS-ester (4 mg, dissolved in 50 μL DMSO; Xi'an Ruixi Biological Technology, China) was added to purified mAb_Nectin-4_ (150 μg, 1 nmol), the mixture was reacted with continuous oscillation for 12 h (4 °C, away form light) to synthesize mAb_Nectin-4_-ICG mixture. The mAb_Nectin-4_-ICG mixture was further purified with PD-10 columns (mobile phase: PBS). The absorption spectrum of samples were detected by UV−vis–NIR spectrometer. The storage stability of mAb_Nectin-4_-ICG was also tested away light till 48 h.

Prepare ICG solution with graded concentrations within PBS (1, 5, 10, 20, 50, 100, 250, 500, and 1000 μg/mL), and detect the optical density (OD) values with an microplate reader. Take the ICG concentrations as the abscissas and the OD value as the ordinates to built the ICG concentration-absorbance standard curve and obtain a regression equation. The standard curve is applied to calculate the ICG concentration in the in mAb_Nectin-4_-ICG solution in further studies.

### Photothermal property of mAb_Nectin-4_-ICG

To explore the photothermal performance of the mAb_Nectin-4_-ICG, 100 μL of mAb_Nectin-4_-ICG aqueous solution containing different ICG concentrations [0 (water), 1, 5, 10, 20, and 30 μg/mL] placed in separate plate (Corning™, Stripwell Plates, 1 × 8 strips, 0.36 mL) was irradiated with 808 nm laser (Honglan Electronic Technology Co., Ltd. Beijing, China) with multiple power densities (0.1, 0.3, 0.5, 0.8, 1.0, and 1.5 W/cm^2^). The solution temperature was detected by a handheld infrared thermal detector (FLIR^®^, E8xt, FLIR Systems, Inc. USA). Photothermal stability was explored following 4 laser on–off cycles: mAb_Nectin-4_-ICG solution (20 μg/mL) was treated with laser irradiation (1 W/cm^2^, 3 min), then the laser was turned off for cooling solution to room temperature.

### Biological safety and in vitro targeting of mAb_Nectin-4_-ICG

Seed MDA-MB-468 cells in a 96-well plate (density: 1.0 × 10^4^) in 100 μL complete L-15 medium. After overnight cultivation, switch to serum-free medium containing mAb_Nectin-4_-ICG/free ICG with different ICG doses (1, 5, 10, 20, 30, 40, 50, 100, and 200 μg/mL) and continue incubating for 4 h. Rinsed the cells by PBS twice, and co-incubate the cells with 1 × Cell counting kit-8 (CCK-8) solution for another 4 h. Utilizing a multi-functional microplate reader to detect the absorbance at 450 nm and calculate the cell viability (n = 5).

Following same seeding conditions, after complete attachment, co-incubate MDA-MB-468 cells with mAb_Nectin-4_-ICG or free ICG (20 μg/mL, 100 μL/well; within serum-free medium) for 4 h. The treated cells were flushed using PBS twice, then imaged with an IVIS Spectrum imaging system (Bruker, Germany; excitation/emission of 750/790 nm fliters). For blocking studies, cells were incubated previously with excess mAb_Nectin-4_ for 1 h.

### In vitro PTT

MDA-MB-468 cells (seeded in 96-well plate; 1.0 × 10^4^ cells/well; 100 μL medium; incubated overnight) was applied for in vitro PTT studies. Employ a standard CCK-8 assay to test the cell viability: (1) For ICG concentration-dependent study, cells were treated with serum-free medium containing graded ICG doses (0, 1, 5, 10, 20, and 30 μg/mL) in either mAb_Nectin-4_-ICG or free ICG and continue incubated for 4 h. Serum-free L-15 medium was switched after washing cells with pre-cooling PBS twice, and then the cells were exposed to the laser irradiation (1.0 W/cm^2^, 10 min); (2) For power density-dependent study, all cells were incubated with mAb_Nectin-4_-ICG/free ICG (containing 20 μg/mL ICG) and the irradiation was performed with graded power densities (0.1, 0.3, 0.5, 0.8, 1.0, and 1.5 W/cm^2^; 10 min); and (3) For irradiated time-dependent study, cells were incubated with mAb_Nectin-4_-ICG/free ICG (20 μg/mL ICG), following by laser irradiation with 1.0 W/cm^2^ power for different durations (0, 2, 4, 6, 8, and 10 min). In vitro PTT on MCF-7 cells was also performed (ICG concentration: 20 μg/mL; laser intensity: 1 W/cm^2^; and irradiated time: 10 min).

Calcein AM/PI staining was also performed for verifying the cellular viability. Seeded MDA-MB-468 cells (1.0 × 10^4^ cells/well in 96-well plates) were co-incubated with mAb_Nectin-4_-ICG or free ICG (20 μg/mL ICG) for 4 h. Then irradiate the cells using 808 nm laser (1.0 W/cm^2^, 10 min). Calcein AM/PI doulbe staining was taken and the FL imaging was performed by microscope.

### In vivo and ex vivo fluorescence imaging

MDA-MB-468 xenograft tumor-bearing mice were administrated intravenously with 200 μL mAb_Nectin-4_-ICG, free ICG (1 mg/kg ICG dose), or saline. Mice were anesthetized by 1% pentobarbital sodium during imaing process. The in vivo fluorescence (FL) imaging at multiple time points (3, 6, 12, 24, 36, and 48 h) was achieved with IVIS Spectrum imaging system with Excitation/Emission of 750/790 nm) and analyzed using Bruker MI software. The ROIs of the tumor area (tumor) and the lower limb muscle (muscle) were delineated and the corresponding FL intensity was obtained. The T/M ratio was calculated as Intensity_tumor_/Intensity_muscle_. After the last scanning time point, all mice were killed to collect the tumors and major organs to conduct the ex vivo FL imaging. The intensity of each organ and tumors were measured for quantification and comparison.

### In vivo PTT for TNBC tumor

The tumor-bearing mice were indiscriminately assigned to 4 groups (① mAb_Nectin-4_-ICG + laser; ② free ICG + laser; ③ saline + laser; and ④ saline; n = 6) and received different treatments. All groups were administrated with 200 μL mAb_Nectin-4_-ICG, free ICG solution (1 mg ICG/kg) or saline via tail veins, respectively. Twenty-four h post injection, ①, ②, and ③ groups were irradiated with 808 nm laser (1.0 W/cm^2^, 5 min) whereas ④ group did not underwent laser therapy. During the irradiation, anesthesia was maintained with isoflurane, and the tumor temperature and the corresponding thermal images were detected. The tumor volume (length and width) and body weight were measured during the 30-day follow-up period. The tumor volume and relative tumor volume were computed as V = D × d^2^/2, and V_R_ = V_X_/V_0_, respectively (D refer the maximum diameter of tumor, and d refer the minor diameter; V_X_ refer the volume on day X, and V_0_ refer the initial tumor volume prior to treatment). On the 30th day, sacrifice all mice and weight the tumor tissues. Moreover, harvest the blood and the selected organs. The hematology tests [white blood cells (WBC), platelets (PLT), red blood cells (RBC), and hemoglobin (HGB)], blood biochemistry tests [alanine transaminase (ALT), aspartate aminotransferase (AST), alkaline phosphatase (ALP), blood urea nitrogen (BUN), and creatinine (CRE)].Hematoxylin and eosin (H&E) staining of selected organs were processed to evaluate the systematic toxicity.

### Statistical analysis

Statistical analysis and charting were conducted employing GraphPad Prism software (version 8.0, USA). The data are described as mean ± standard deviation. *p* < 0.05 was regarded as statistically significant.

## Results

### Expression of Nectin-4

**Fig. 1:**
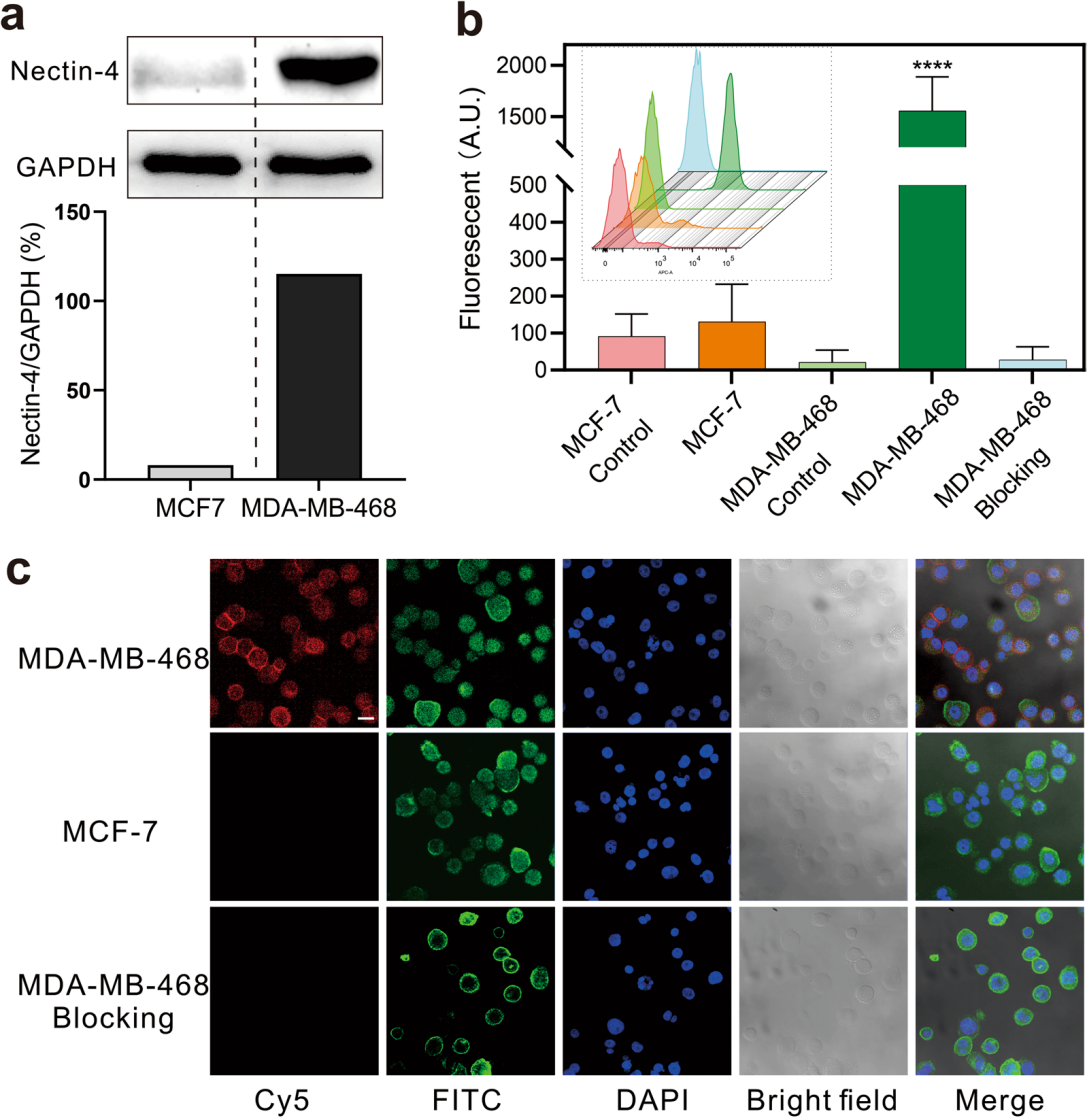
Nectin-4 is overexpressed in MDA-MB-468 TNBC cells. **a** Western blot results of Nectin-4 expression in MDA-MB-468 cells and MCF-7 cells; GAPDH is used as an internal reference. **b** Flow cytometry results (inner subgraph) and fluorescence intensity analysis (n = 3, ****p < 0.0001 versus control). **c** CLMS imaging results, Cy5.5 conjugated mAb_Nectin-4_ (red), FITC-phalloidin (green), and DAPI (blue) were used for Nectin-4 target, cytoskeleton, and nuclear staining, respectively; the scale bar = 20 μm

We first performed western blotting to detect the expression of Nectin-4 in MDA-MB-468 and MCF-7 cells. Overexpression of Nectin-4 was demonstrated in MDA-MB-468 cells (Fig. 1a), but depressed expression was noticed in MCF-7 cells. The results of flow cytometry (Fig. 1b) and CLSM imaging (Fig. 1c) further demonstrated the high cell surface expression of Nectin-4 with high specificity in MDA-MB-468 cells. Basing on these findings, we selected MDA-MB-468 cells as the experimental group whereas MCF-7 as the control group in subsequent studies.

### Synthesis and in vitro targeting ability of ^99m^Tc-HYNIC-mAb_Nectin-4_

**Fig. 2:**
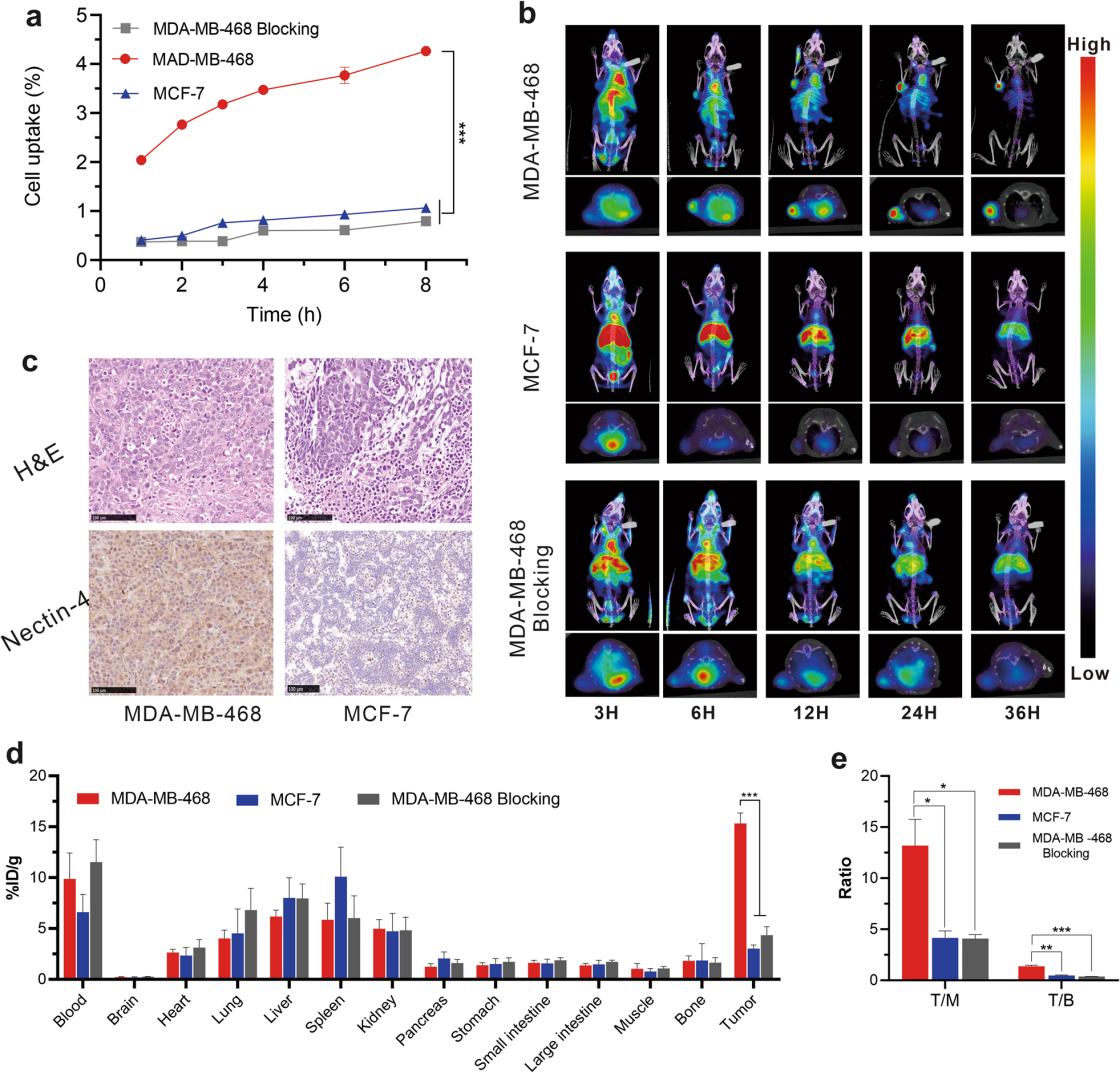
^99m^Tc-HYNIC-mAb_Nectin-4_ has high Nectin-4-targeting in vitro and in vivo. **a** Cellular uptake assays of ^99m^Tc-HYNIC-mAb_Nectin-4_ at 1−8 h (n = 4), ***p < 0.001. **b** ^99m^Tc-HYNIC-mAb_Nectin-4_ SPECT/CT imaging of xenograft tumor-bearing mice at different time points, each subgraph includes MIP (upper) and transaxial (lower) image. **c** The H&E and Nectin-4 immunohistochemistry staining results of xenograft tumor tissues, scale bar = 100 μm. **d** Biodistribution of ^99m^Tc-HYNIC-mAb_Nectin-4_ in different organs and tumors of tumor-bearing mice at 36 h p.i.. ***p < 0.001 (n = 3). **e** The corresponding T/M and T/B ratio from biodistribution, *p < 0.05, ** < 0.01, ***p < 0.001

The radionuclide probe ^99m^Tc-HYNIC-mAb_Nectin-4_ was successfully synthesised with a high radiolabelling yield (73.03 ± 6.95%) and radiochemical purity (95.03 ± 2.40%) (Additional file 1: Fig. S1). To assess the in vitro targeting ability of this radiotracer, a cell uptake assay was conducted. As illustrated in Fig. 2a, MDA-MB-468 cells had specific in vitro ^99m^Tc-HYNIC-mAb_Nectin-4_ uptake with a relatively higher uptake of 2.04 ± 0.09% at 2 h. The uptake rate gradually increased over time with the highest uptake of 4.27 ± 0.09% at 8 h (Fig. 2a). In comparison, the control MCF-7 cells (1.07 ± 0.15%) and blocked MDA-MB-468 cells (0.79 ± 0.03%) had significantly lower uptake at 8 h. These findings indicate that ^99m^Tc-HYNIC-mAb_Nectin-4_ could target Nectin-4-positive cells with eminent affinity and specificity.

### SPECT/CT and biodistribution

MicroSPECT/CT images of tumor-bearing mice were collected to explore the in vivo tumor-targeting of ^99m^Tc-HYNIC-mAb_Nectin-4_ (Fig. 2b). In the experimental group, the tumor radioactivity could be recognised at 3 h p.i. and was gradually enhanced with time. Prominent radioactivity at tumor sites was observed with an excellent target-background contrast at 24 and 36 h p.i. In addition, relatively obvious radioactivity was also noted in some extra-tumoral regions such as the heart and liver at early time points, which decreased with time. In comparison, the tumors of the control and blocked groups had obviously lower ^99m^Tc-HYNIC-mAb_Nectin-4_ uptake, whereas normal liver had continuous radioactivity accumulation. We also performed a semi-quantitative analysis to compare ^99m^Tc-HYNIC-mAb_Nectin-4_ uptake in different groups, and the results were consistent with the imaging findings (Additional file 1: Fig. S2). Moreover, the immunohistochemical staining results (Fig. 2c) confirmed the overexpression of Nectin-4 in MDA-MB-468 TNBC xenograft tumors, significantly superior to that of MCF-7 tumors. These findings further verified the SPECT imaging results.

The results of the biodistribution study directly demonstrated the different uptake by xenograft tumors and organs in each group (Fig. 2d). The ^99m^Tc-HYNIC-mAb_Nectin-4_ accumulation of MDA-MB-468 xenograft tumor (15.32 ± 1.04% ID/g) was substantially greater than that of MCF-7 xenografts (3.02 ± 0.20% ID/g, *p* < 0.001) and blocked MDA-MB-468 groups (4.33 ± 0.48% ID/g, *p* < 0.001), respectively. Additionally, the MDA-MB-468 group mice exhibited superior T/M and T/B ratio compared with the MCF-7 and blocked MDA-MB-468 groups (Fig. 2e). Collectively, these results confirmed that ^99m^Tc-HYNIC-mAb_Nectin-4_ can bind to Nectin-4-positive tumors in vivo with an excellent detection capability, high specificity, and good tumor retention.

### Synthesis and characterisation of mAb_Nectin-4_-ICG

**Fig. 3:**
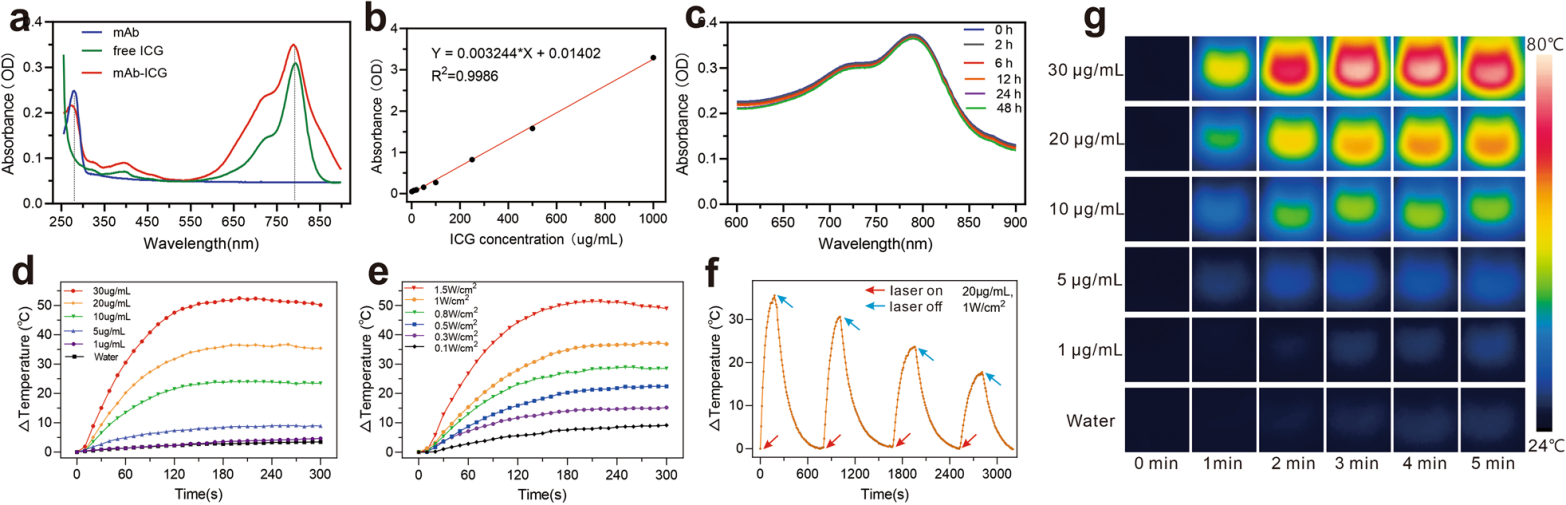
The optical characterization and photothermal property of mAb_Nectin-4_-ICG. **a** The UV − vis absorbance spectra. **b** ICG standard concentration-absorbance curve at 790 nm. **c** Stability test of mAb_Nctin-4_-ICG at 0–48 h. **d** Photothermic heating curves of mAb_Nectin-4_-ICG solutions containing different ICG concentrations with laser irradiation (1.0 W/cm^2^). **e** Photothermic heating curves of a mAb_Nectin-4_-ICG (20 μg/mL ICG concentration) solution irradiated with 808 nm laser following graded power densities. **f** Photothermal stability of mAb_Nectin-4_-ICG solution over 4 repeated laser on (20 μg/mL ICG concentration, 1.0 W/cm^2^, 3 min) and laser off cycles. **g** The corresponding thermal images for **d**

Encouraged by the desirable Nectin-4-targeted SPECT/CT imaging results, we further designed an mAb_Nectin-4_-based fluorescent probe (mAb_Nectin-4_-ICG) as a PTA for TNBC xenograft PTT. The characteristic absorbance peaks of mAb_Nectin-4_-ICG was observed at 280 nm and 790 nm, which were is similar to that of mAb_Nectin-4_ and free ICG, respectively (Fig. 3a). These findings prove the successful synthesis of mAb_Nectin-4_-ICG and there was no significant effect on the optical features of ICG. Therefore, we further determined the regression equation (Y = 0.003244 * X + 0.01402) basing on the 790 nm-absorbance value of different free ICG concentrations (Fig. 3b), which was used to calculate the concentration of ICG in mAb_Nectin-4_-ICG solution. In dark conditions, the peaks of mAb_Nectin-4_-ICG did not decrease significantly with time (Fig. 3c), indicating that this PTA had favourable stability.

### Photothermal properties of mAbNectin_-4_-ICG

To investigate the photothermal conversion effect of mAb_Nectin-4_-ICG, we recorded the temperature rise of mAb_Nectin-4_-ICG under laser exposure at graded ICG concentrations or power densities. First, mAb_Nctin-4_-ICG solution with different ICG concentrations [0 (water), 1, 5, 10, 20, and 30 μg/mL] was exposed to 808 nm laser at 1 W/cm^2^ power density. The results showed that solution temperature increased with ICG concentration (Fig. 3d and g). While the ICG concentration reached 20 μg/mL, solution temperature rapidly elevated within 2 min after irradiation, which exceeded 55 °C (starting temperature = room temperature: 25 °C, △T > 30 °C) and continued to rise slowly. This finding indicated that the cell damage temperature threshold (42 °C) could be reached when the ICG concentration exceeded 20 μg/mL. As a comparison, temperature increase of water did not exceed 5 °C.

Then, the mAb_Nectin-4_-ICG (20 μg/mL) solutions were exposed to 808 nm laser using graded power densities (0.1, 0.3, 0.5, 0.8, 1, and 1.5 W/cm^2^) to investigate the photothermal performance. As displayed in Fig. 3e, the photothermal conversion property of mAb_Nectin-4_-ICG solution significantly enhanced with the power density. Solution temperature elevated to 47.4 °C at 5 min when irradiation was performed using 0.5 W/cm^2^; as the power reached 1.5 W/cm^2^, temperature could be elevated to 73.9 °C.

Four laser on–off cycles were taken to investigate the photothermal stability. As shown in Fig. 3f, compared with the previous cycle, the photothermal performance mildly deteriorated after each irradiation. However, the peak temperature of the fourth cycle could also exceed 42 °C (△T = 18 °C), indicating that as a PTA, mAb_Nectin-4_-ICG had good stability and reproducibility**.**

### In vitro biological safety and targeting of mAb_Nectin-4_-ICG

**Fig. 4:**
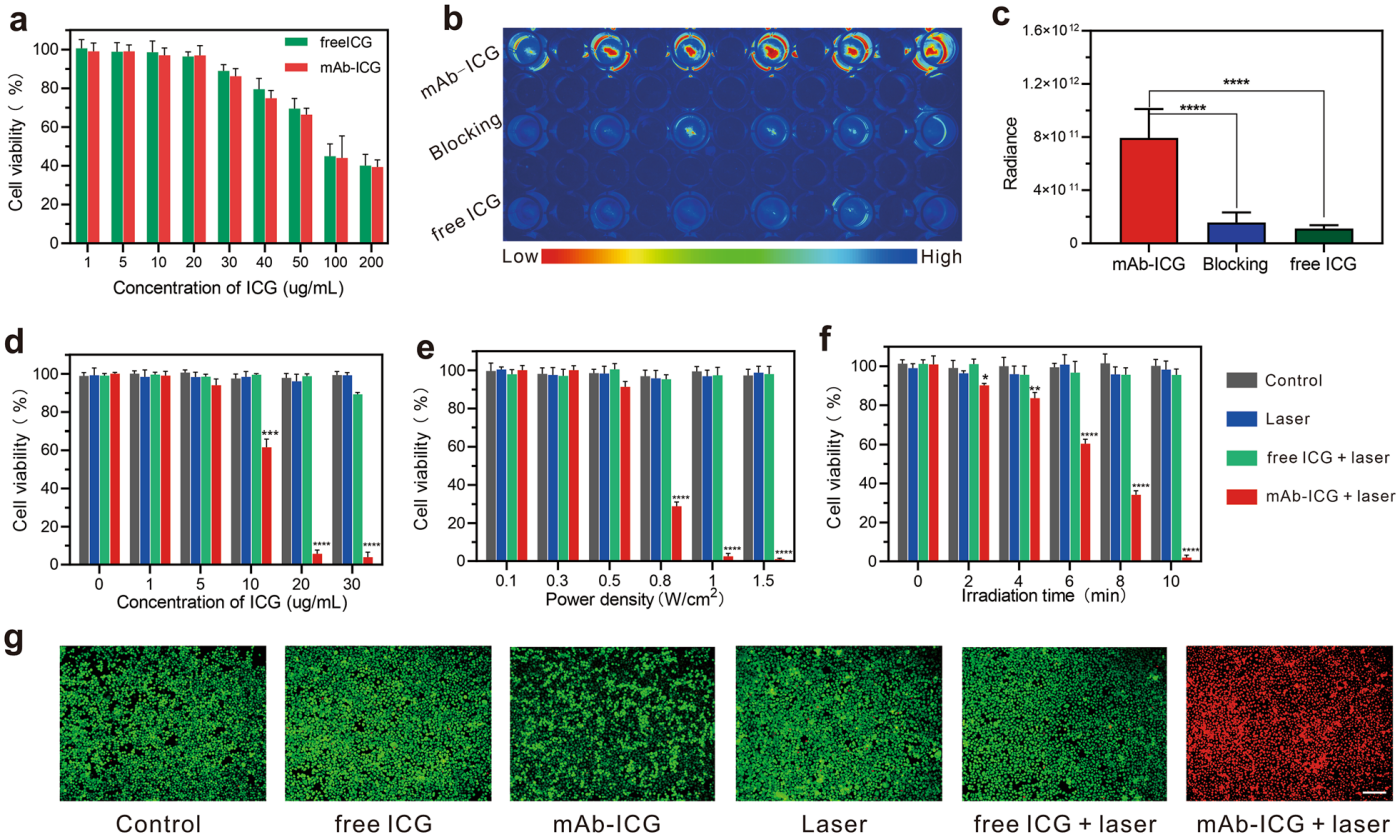
In vitro biological safety, targeting and PTT effects of mAb_Nectin-4_-ICG. **a** Cytotoxicity of mAb_Nectin-4_-ICG and free ICG containing different ICG concentrations on MDA-MB-468 cells (n = 5). **b**, **c** In vitro cell targeting study of mAb_Nectin-4_-ICG and corresponding fluorescence quantitative analysis. **d**–**f** The viability of MDA-MB-468 cells after different treatments. ICG concentration-dependent PTT (**d**) was performed with 1.0 W/cm^2^ power for 10 min following cells treated with graded ICG concentrations; power density-dependent PTT (**e**) was performed at graded laser power densities for 10 min following cells treated with mAb_Nectin-4_-ICG/free ICG (20 μg/mL ICG); irradiated time-dependent study (**f**) was performed following cells treat with mAb_Nectin-4_-ICG/free ICG (20 μg/mL ICG), and then the PTT was conducted at 1.0 W/cm^2^ power density for different times ranging 0–10 min; *p < 0.05, **p < 0.01, ***p < 0.001, ****p < 0.0001 compared to control. **g** Celluar FL images of Calcein AM/PI staining following different treatment, the green signal refers to viable cells whereas the red signal refers to dead cells, scale bar = 200 µm

The in vitro cytotoxicity of mAb_Nectin-4_-ICG was examined with CCK-8 assay (Fig. 4a). According to our results, cell viability exceeded 90% if the ICG concentration lower than 20 μg/mL. However, when the ICG concentration greater than 30 μg/mL, the cell viability was less than 90% and gradually decreased as the ICG concentration elevated. The above results indicated that when the ICG concentration does not exceed 20 µg/mL, mAb_Nectin-4_-ICG had only low cytotoxicity, which provided a basis for subsequent in vitro experiments.

For verifying the in vitro targeting of mAb_Nectin-4_-ICG, MDA-MB-468 cells were incubated with this PTA and then FL imaging was performed. As demonstrated in Fig. 4b, cells co-incubated with mAb_Nectin-4_-ICG had obvious FL uptake, whereas only scant fluorescence signal was observed in the blocked cells and free ICG-treated cells. This proves that through the specific binding of antigen and antibody, the PTA could target the cells with high Nectin-4 expression. Semi-quantitative analysis of FL intensity (Fig. 4c) also support above results (*p* < 0.0001).

### In vitro PTT of mAbNectin-4-ICG

After MDA-MB-468 cells were incubated with mAb_Nectin-4_-ICG/free ICG, in vitro thermal ablation efficiency was analysed with laser irradiation at different ICG concentrations, power intensities, and irradiation time. In general, as displayed in Fig. 4d–f, free ICG, mAb_Nectin-4_-ICG only, laser only, and free ICG plus laser showed negligible cytotoxicity. The cell viability of each group was above 90% free ICG only, mAb_Nectin-4_-ICG only, and free ICG plus laser groups at an ICG concentration of 30 μg/mL had a certain degree of cell lethality (approximately 10%), which was considered to be the inherent toxicity of free ICG/mAb_Nectin-4_-ICG at this high ICG concentration (Fig. 4a). As expected, the mAb_Nectin-4_-ICG plus laser group exhibited an obvious response to laser irradiation, and the increase in ICG concentration, laser power density, and irradiated time exacerbated cell death. Furthermore, more than 90% of MDA-MB-468 cells were destroyed when ICG concentration and power density reached 20 μg/mL and 1 W/cm^2^ (irradiation time = 10 min), respectively. The effectiveness of in vitro PTT on MCF-7 cells was also evaluated, the results demonstrated that mAb_Nectin-4_-ICG mediated PTT failed to provide effective cell killing on cells with low Nectin-4 expression (Additional file 1: Fig. S3).

FL microscopy image analysis of MDA-MB-468 cells following calcein-AM (living cells, green signal) and PI (dead cells, red signal) staining further affirmed these results (Fig. 4g). Diffuse deep red FL signals were observed in cells treated with mAb_Nectin-4_-ICG plus 808 nm laser, indicating that the cells were almost completely dead. In contrast, only scattered red-stained cells were observed in the diffuse bright green staining field, proving that cell death was negligible. These results suggest that mAb_Nectin-4_-ICG promoted ICG accumulation and led to prominent thermal ablation efficiency.

### FL imaging

**Fig. 5:**
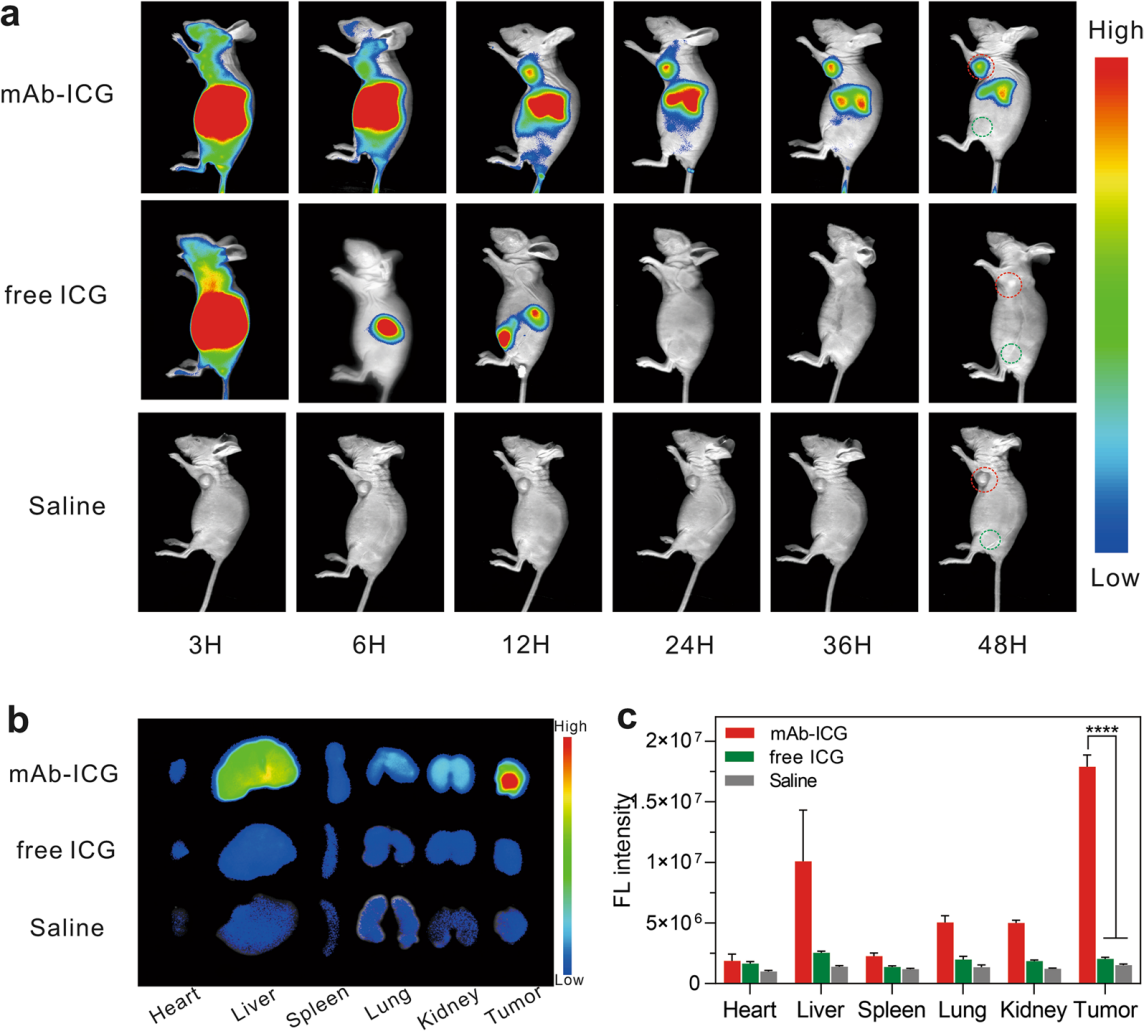
The mAb_Nectin-4_-ICG has excellent tumor-specific targeting and retention in Nectin-4-overexpression tumor in vivo. **a** FL imaging for MDA-MB-468 xenografts tumor-bearing mice administrated with mAb_Nectin-4_-ICG, free ICG or saline. Red circles indicates the tumor sites and green circles indicates the normal muscles (n = 3). **b** Ex vivo FL imaging for isolated organs/tumors at 48 h p.i. **c** Semi-quantitative assays of FL signal intensity for **b**, ****p < 0.0001 (n = 3)

To explore the in vivo tumor-targeting property of mAb_Nectin-4_-ICG and the best time window for PDT, MDA-MB-468 TNBC tumor-bearing mice administrated with mAb_Nectin-4_-ICG, free ICG, or saline underwent in vivo FL scanning at various time points (Fig. 5a). The FL signal of mAb_Nectin-4_-ICG was chiefly accumulated in the liver initially and the hepatic fluorescence gradually decreased, whereas a relatively obvious tuomor FL signal was detected at 12 h p.i., which continuously elevated over time and peaked at 24 h p.i. In comparison, no prominent tumor FL signal was captured in the mice injected with free ICG and saline. The FL quantification of xenograft tumor ROIs (Additional file 1: Fig. S4a) and tumor/muscle (T/M) semi-quantitative (Additional file 1: Fig. S4b) analysis also exhibited the same trend.

Ex vivo FL imaging at 48 h p.i. (Fig. 5b) was also performed. Prominent mAb_Nectin-4_-ICG tumor uptake was observed, with partially retained by the liver and kidney in experimental group. Subtle FL signals were detected in the tumor and in each organ in the free ICG group, almost similar to that of the saline group (background signal), suggesting that free ICG is rapidly metabolised in the body because of its small molecular weight and is basically completely eliminated by 48 h. The corresponding FL intensity analysis further certify the findings (Fig. 5c). These findings showed that mAb_Nectin-4_-ICG has excellent tumor-specific targeting and long-term retention, which provides a basis for in vivo PTT. Hence, 24 h p.i. was determined to be the optimal PTT therapy time.

### In vivo anti-tumor studies

**Fig. 6:**
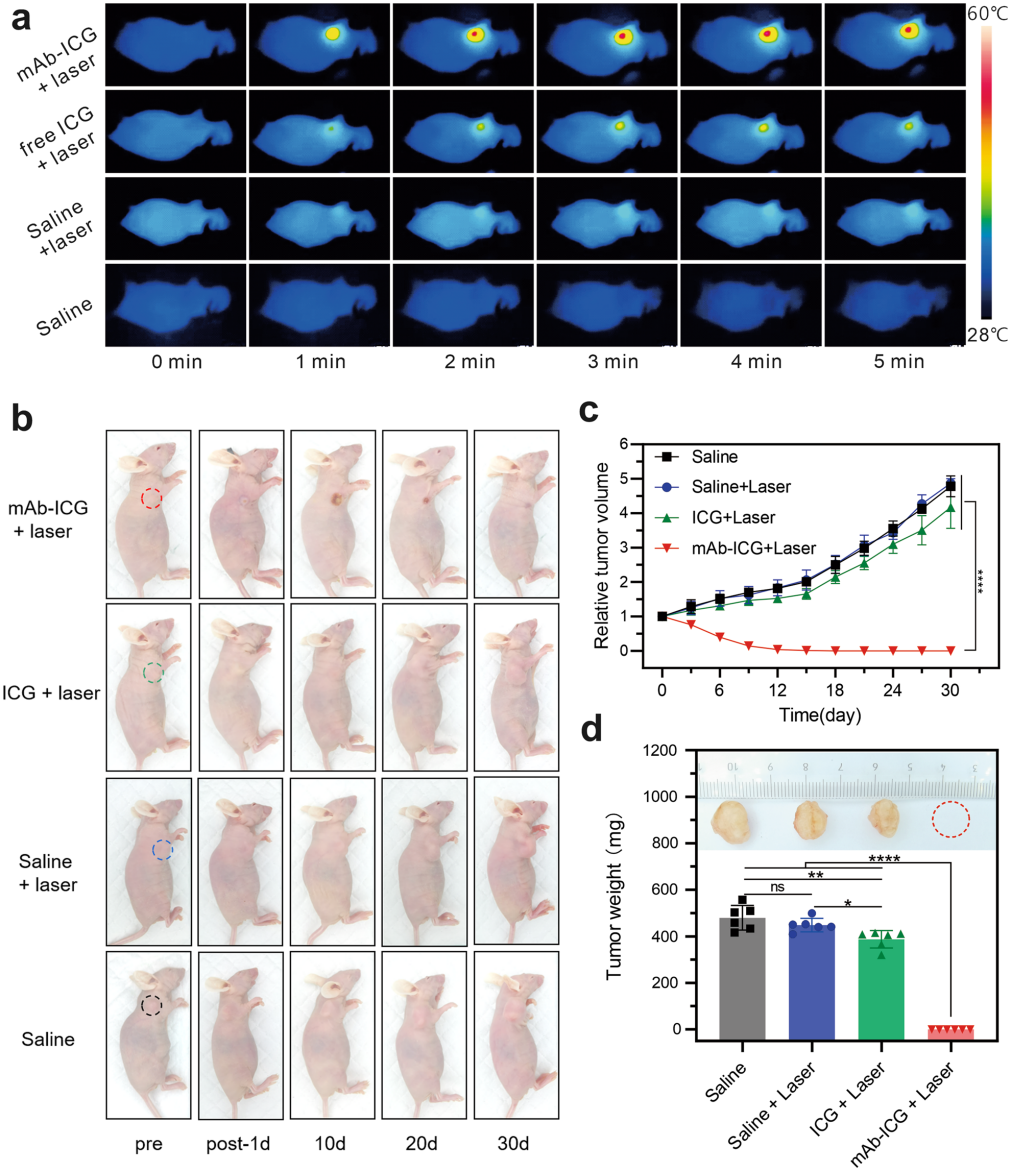
The mAb_Nectin-4_-ICG mediated PTT induced efficient anti-tumor effect. **a** Infrared thermal imaging at the tumor sites at different time intervals. **b** Representative photos of tumors at different days post treatment. **c** Relative tumor growth curves in each group. **d** The tumor weights on the 30th day post treatment (*ns* not significant; *p < 0.05, **p < 0.01, and ****p < 0.0001)

In vivo PTT effect mediated by mAb_Nectin-4_-ICG was further investigated using MDA-MB-468 tumor-bearing mice. As shown in Fig. 6a and Additional file 1: Fig. S5, upon 808 nm laser exposure, the tumor temperature increased rapidly within 2 min (△T = 15 °C) in mAb_Nectin-4_-ICG + laser group mice, which exceeded the damage threshold (42 °C; nude mouse basal body T approximately 30 °C) and would result in irreparable tissue damage. In comparison, after laser irradiation, the elevated temperature in tumor sites of the free ICG + laser and saline + laser groups did not exceed 10 °C and 5 °C, respectively (neither reached the damage threshold value), demonstrating either free ICG-mediated irradiation or laser alone failed to provoke hyperthermia for effective therapy. In mice without laser irradiation, there was almost no change in the temperature of the tumor site, and even a slight decrease was recorded, which may be attributed to the continued anaesthesia.

Representative images of mice exhibiting PTT-induced anti-tumor effects are displayed in Fig. 6b. After laser exposure, the skin of the tumor site of the mAb_Nectin-4_-ICG + laser group blanched and turned hard. Then, scabs gradually appeared on the irradiated site, which began to fall off and form keloids around the 10th day. The relative tumor volume–time curves (Fig. 6c) showed a negative growth trend in the mAb_Nectin-4_-ICG + laser group, and the tumors on the 12th day were almost unmeasurable (volume recorded as 0 mm^3^), suggesting the tumors were completely ablated. In contrast, the tumors of the free ICG + laser, saline + laser, and saline only groups did not change significantly in shape and colour after treatment, and no significant suppression of tumor growth was observed (Fig. 6b, c). On the 30th day after receiving treatment, the tumors of each group were harvested and weighed (Fig. 6d). Because the tumor volume could not be measured, the weight of tumors from the mAb_Nectin-4_-ICG + laser group was recorded as 0.00 ± 0.00 mg, which was significantly lower than the remaining three groups (free ICG + laser group: 387.37 ± 37.56 mg; saline + laser group: 448.63 ± 29.14 mg; and saline group: 479.67 ± 52.96 mg, *p* < 0.0001). In summary, only PTT mediated by mAb_Nectin-4_-ICG, which has both tumor-specific targeting and photothermal conversion abilities, can achieve substantial tumor heating and result in efficient and significant tumor growth oppression.

**Fig. 7:**
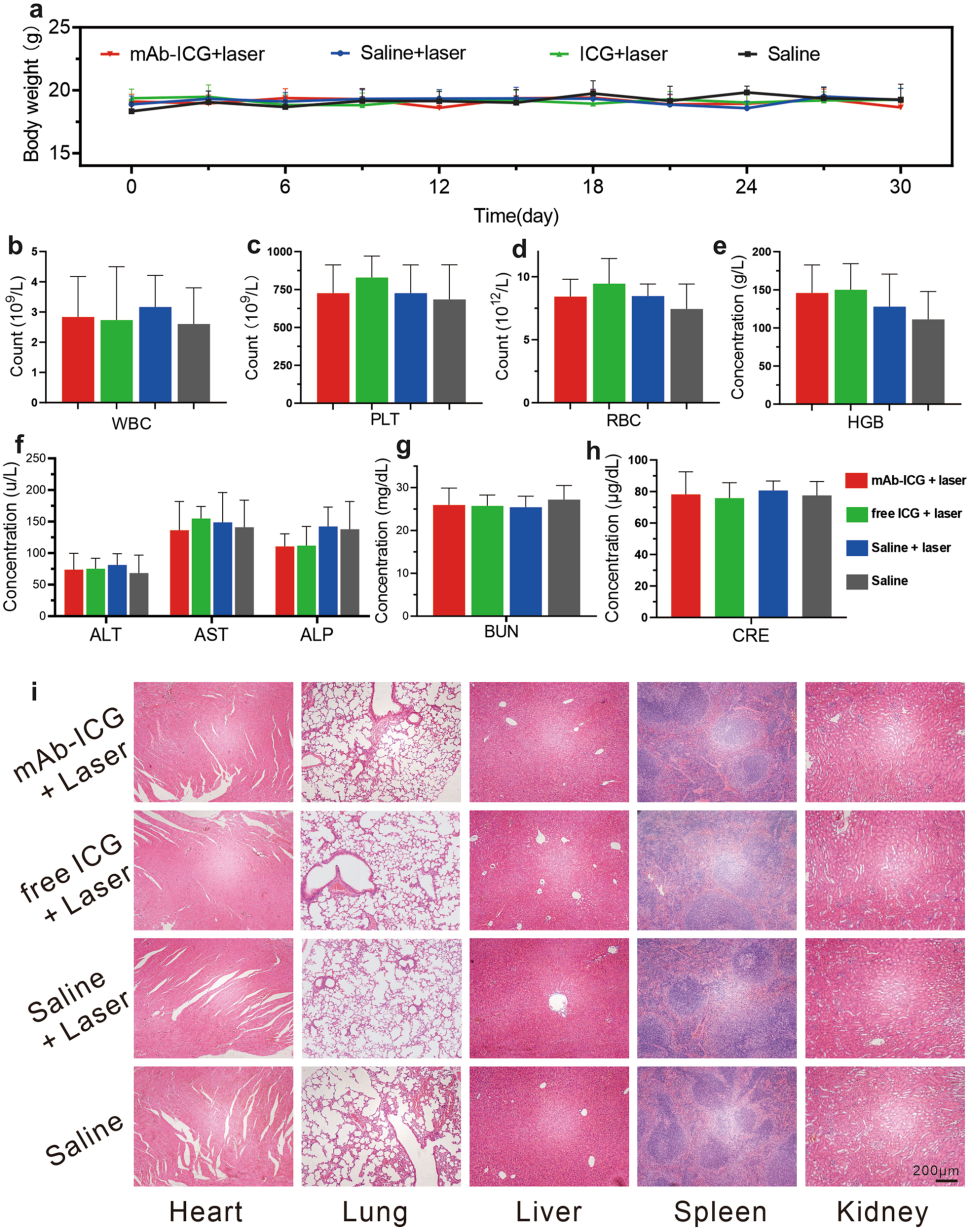
mAb_Nectin-4_-ICG mediated PTT has no significant systemic toxicity. **a** The body-weight-change curves in 30-day duration of different groups. **b**–**h** Blood examination result (n = 6). **i** H&E staining of selected organs, scale bars = 200 μm

No death or significant weight fluctuations were noticed in each groups during this 30-day observation period (Fig. 7a), suggesting negligible systemic side effects. To further explore the biological safety of PTT, the blood and selected organs were harvested on the 30th day post therapy. The results of blood examinations in each group were all within the normal range (Fig. 7b–h). On the HE-stained slices of selected organs, no inflammation or necrosis was evident in each groups (Fig. 7i). The findings indicated that mAb_Nectin-4_-ICG-mediated PTT induced no significant side effects or toxicity.

## Disccusion

In this study, using mAb against Nectin-4 as a carrier, we designed a radioprobe, ^99m^Tc-HYNIC-mAb_Nectin-4_, which exhibited considerable Nectin-4-specific targeting in vitro and in vivo. This probe-mediated SPECT/CT imaging could achieve TNBC diagnosis and visualization of Nectin-4 expression, which provided a classification basis for Nectin-4-related treatments. We also developed an antibody-based PTA, mAb_Nectin-4_-ICG, which presented favourable biocompatibility and photothermal performance without obvious biotoxicity. Through FL imaging, we further determined the distribution of mAb_Nectin-4_-ICG in vivo and the optimal treatment time window. Excitingly, mAb_Nectin-4_-ICG-mediated PTT for TNBC xenografts demonstrated significant tumor suppression, indicating that tumor-targeting antibody can accurately deliver ICG to the tumor site, resulting in enhanced PTT effects.

Recent investigations revealed that Nectin-4 is involved in several facets of tumor progression, including proliferation, angiogenesis, and epithelial-to-mesenchymal transition in various cancers, including breast cancer [19]. Drug resistance, distant metastasis, tumor relapse, and poor prognosis are also related to the Nectin-4 overexpression [19]. Currently, a variety of therapeutic strategies against Nectin-4 are used for cancer treatment, including bioactive/synthetic compounds (e.g., nanoquinacrine) [38], antibody–drug conjugates (ADCs) [39], and oncolytic viruses [40]. Among them, multiple clinical trials have shown that ADCs are more powerful than traditional drugs in suppressing tumors [41, 42]. Enfortumab vedotin, as a promising ADC for metastatic urothelial carcinoma, became the first FDA-approved ADC drug [43]. Therefore, Nectin-4 has potential to be the most promising therapeutic target and prognostic biomarker for Nectin-4-overexpressing cancer, including TNBC [17]. In-depth understanding of this biomarker is beneficial to the study of Nectin-4-related pathophysiological processes in cancer. Our study reported that Nectin-4-positive TNBC tumors can have intensive radioactivity accumulation on ^99m^Tc-HYNIC-mAb_Nectin-4_ SPECT imaging, which agreed the findings from previous publication [44]. Campbell et al. [44] used Zr-89-labelled Nectin-4-targeted antibody for PET imaging, obtained a longer imaging window (6 days). Considering the popularity and accessibility of a single-photon radionuclide, the ^99m^Tc-SPECT imaging pair also has clinical translation potential for non-invasive evaluation of Nectin-4 status without relying on invasive biopsy.

Notably, previous studies have shown some non-negligible shortcomings in tumor radioimmunoimaging based on full-length antibodies; that is, slow blood clearance, long imaging time (takes hours or even days to achieve the best distribution in vivo), and high accumulation in non-target tissues including the liver [25, 45]. However, our serial SPECT images showed that although the hepatic radioactivity of the MDA-MB-468 group in the early stages (prior 12 h p.i.) provided a relatively higher background, its uptake was significantly reduced with time, obviously differring from the MCF-7 control group and the blocked MDA-MB-468 group (continuous hepatic accumulation). This may be attributable to the excellent targeting and high affinity of the antibody, which caused it to continuously and highly accumulate in the tumor, resulting in decreased antibody circulating in the blood, and thus the activity of the liver was relatively reduced. Hence, in addition to the advantages of the target biomarker per se, such as high expression in tumors and low/no expression in normal organs, antibodies with high specificity and affinity have benefits in providing a low imaging background. Moreover, most of the current clinical SPECT or PET scanners have been integrated with CT or MR (such as PET/CT, SPECT/CT, and PET/MR), which promote a valuable integration of tumor function information and morphological parameters and contribute to precision diagnosis.

Considering that primary breast cancer and lymph node metastasis (axillary lymph nodes are usually most susceptible to being involved) are located at superficial sites, it is easier to perform laser irradiation treatment compared with deep tumors, and the PTT strategy has been reported in clinical trials [36]. Encouraged by the results of Nectin-4 visualization by SPECT/CT and FL imaging, and on the basis of the superior NIR features of ICG (low auto-fluorescence, preferable tissue penetration, and low toxicity) [46], we designed an ICG-modified antibody conjugate, mAb_Nectin-4_-ICG, as a PTA to conduct PTT for TNBC. Our results demonstrated that this PTA had a good photothermal effect, and exact control of PTT could be achieved by regulate the ICG concentration, power density and irradiation time. Following an mAb_Nectin-4_-ICG-mediated NIR-PTT regimen, MDA-MB-468 xenograft tumor growth was obviously suppressed and completely ablated. Two reasons may explain such a powerful anti-tumor effect. One is the superb targeting and affinity of mAb_Nectin-4_, which has been verified on SPECT and FL imaging, resulting in the accurate delivery of ICG to the tumor site by antibody. Hence, intense tumor ICG accumulation could response to the laser to generate hyperthermia and provoking irreversible cellular membrane damage [47]. The second reason may originate from the tumor per se. MDA-MB-468 xenograft tumor growth is relatively slow compared with other tumors, and the initial pre-treatment tumor volume in our protocols was relatively small. In this case, the laser could easily cover the tumor entirely and cause complete ablation. Certainly, in addition to the above-mentioned aspects, under 808 nm laser exposure, ICG could also produce reactive oxygen species to achieve PTT [31]. This synergistic anti-tumor effect cannot be ignored.

While pursuing a powerful tumor killing efficiency, PTT brings an unavoidable problem. When the tumor is irradiated and ablated, the skin is also exposed to the laser and causes certain damage, such as oedema, peeling, and crusting, which will eventually form scars [36]. In addition, hypertrophic scars and keloid formation can occur after surgery or after other invasive treatments such as the laser therapy discussed here, which results in a functional and sensory disorder at the scar sites. Moreover, the skin damage after treatment may cause psychological issues for patients who are concerned about their appearance. Thus, how to balance effective high power-guided treatment and skin damage is a major challenge in PTT. Some studies focused on developing novel PTAs or modifying existed PTAs that could induce higher photothermal conversion efficiency and potentially lessen skin damage [48, 49]. Combining other therapies with PTT to produce synergistic effects also provides inspiration for the optimisation of PTT [50, 51].

In addition to the ADC therapy that has been used in the clinic, other Nectin-4-targeted theranostic strategies have translatory potential. Various dyes, including ICG, have been used in NIR imaging-guided surgery in pre-clinical studies and clinical practice [52, 53]. The PTA designed here (mAb_Nectin-4_-ICG) could be combined with the NIR imaging system to undoubtedly provide an intraoperative navigation platform for the precise resection of Nectin-4-overexpressing tumor tissues (primary or metastatic tissue). Further experimental data is certainly required to broaden the application spectrum of this PTA probe. Moreover, the radionuclide theranostic strategy mediated by Lu-177-labeled antibody is also worthy of attention [54, 55]. All-in-all, anti-Nectin-4 antibody was shown to be a theranostic carrier, providing an open platform for tumor visualization, screening, and treatment guidance.

## Conclusions

In conclusion, ^99m^Tc-HYNIC-mAb_Nectin-4_ SPECT/CT imaging is useful for delineating Nectin-4 expression in TNBCs and promotes precision diagnosis and classification. Upon stratification by Nectin-4 targeted imaging, mAb_Nectin-4_-ICG-mediated PTT is an up-and-coming remedy for Nectin-4-positive tumors. Integrating the promising SPECT probe and effective PTA as a Nectin-4-targeted theranostic pair may contribute to improve the TNBC patients’ management.

### Supplementary Information

**Additional file 1: Figure S1.** The radiolabelling yield (**a** prior PD-10 purification) and radiochemical purity (**b** post PD-10 purification) of ^99m^Tc-HYNIC-mAb_Nectin-4._ **Figure S2.** The tumor-muscle ratio of radioactivity in xenograft tumor-bearing mice on microSPECT/CT images at different time points, n = 3. **Figure S3.** The viabilities of MCF-7 cells after different treatments, n = 5. **Figure S4.** The fluorescence quantification of the xenograft tumor ROIs (**a**) and the tumor-muscle ratio of FL intensity (**b**) in xenograft-bearing mice on in vivo FL images at different time points, n = 3, ***p < 0.001, ****p < 0.0001. **Figure S5.** The temperature–time curves of xenograft tumor sites during different treatment.

## Abbreviations

TNBC: Triple-negative breast cancer
ER: Estrogen receptor
PR: Progesterone receptor
HER2: Human epidermal growth factor receptor 2
SPECT: Single photon emission computed tomography
PTA: Photothermal agent
NIR: Near infrared
ICG: Ndocyanine green
FDA: Food and Drug Administration
PDT: Photodynamic therapy
PTT: Photothermal therapy
FBS: Fetal bovine serum
SDS-PAGE: Sodium dodecyl sulfate polyacrylamide gel electrophoresis
PVDF: Polyvinylidene fluoride
mAb_Nectin-4_: Anti-Nectin-4 monoclonal antibody
GAPDH: Glyceraldehyde 3‑phosphate dehydrogenase
PBS: Phosphate buffer saline
CLSM: Confocal laser scanning microscopy
SHNH: Succinimidyl 6-hydraziniumnicotinate hydrochloridem
ITLC: Instant thin layer chromatography
SPECT/CT: Single photon emission computed tomography/computed tomography
ROI: Region of interest
MIP: Maximum intensity projection
OD: Optical density
CCK-8: Cell counting kit-8
FL: Fluorescence
WBC: White blood cells
PLT: Platelets
RBC: Red blood cells
HGB: Hemoglobin
ALT: Alanine transaminase
AST: Aspartate aminotransferase
ALP: Alkaline phosphatase
BUN: Blood urea nitrogen
CRE: Creatinine
H&E: Hematoxylin and eosin
ADC: Antibody-drug conjugate
PET: Positron emission tomography
