# Correction: Nectin-4-targeted immunoSPECT/CT imaging and photothermal therapy of triple-negative breast cancer

**DOI:** 10.1186/s12951-023-02096-7

**Published:** 2023-09-23

**Authors:** Fuqiang Shao, Zhidi Pan, Yu Long, Ziyang Zhu, Kun Wang, Hao Ji, Ke Zhu, Wenyu Song, Yangmeihui Song, Xiangming Song, Yongkang Gai, Qingyao Liu, Chunxia Qin, Dawei Jiang, Jianwei Zhu, Xiaoli Lan

**Affiliations:** 1 Department of Nuclear Medicine, Union Hospital, Tongji Medical College, Huazhong University of Science and Technology, No. 1277 Jiefang Ave, Wuhan, 430022 China; 2 Hubei Province Key Laboratory of Molecular Imaging, Wuhan, 430022 China; 3 Department of Nuclear Medicine, Zigong First People’s Hospital, Zigong Academy of Medical Sciences, Zigong, 643000 China; 4 Engineering Research Center of Cell & Therapeutic Antibody, Ministry of Education, School of Pharmacy, Shanghai Jiao Tong University, 800 Dongchuan Road, Shanghai, 200240 China; 5 Jecho Laboratories, Inc., Frederick, MD 21704 USA; 6 Jecho Biopharmaceuticals Co., Ltd., Tianjin, 300467 China; 7 Key Laboratory of Biological Targeted Therapy, The Ministry of Education, Wuhan, 430022 China

**Correction: Journal of Nanobiotechnology (2022) 20:243** 10.1186/s12951-022-01444-3

Following publication of the original article [1], the authors identified an error in Figure. 4g, Figure. 6b, and Figure. 7i. The correct figures are given below.
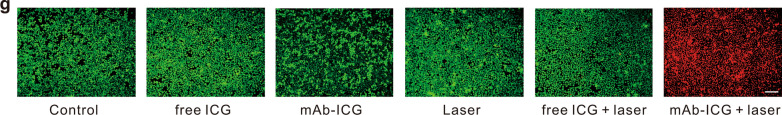



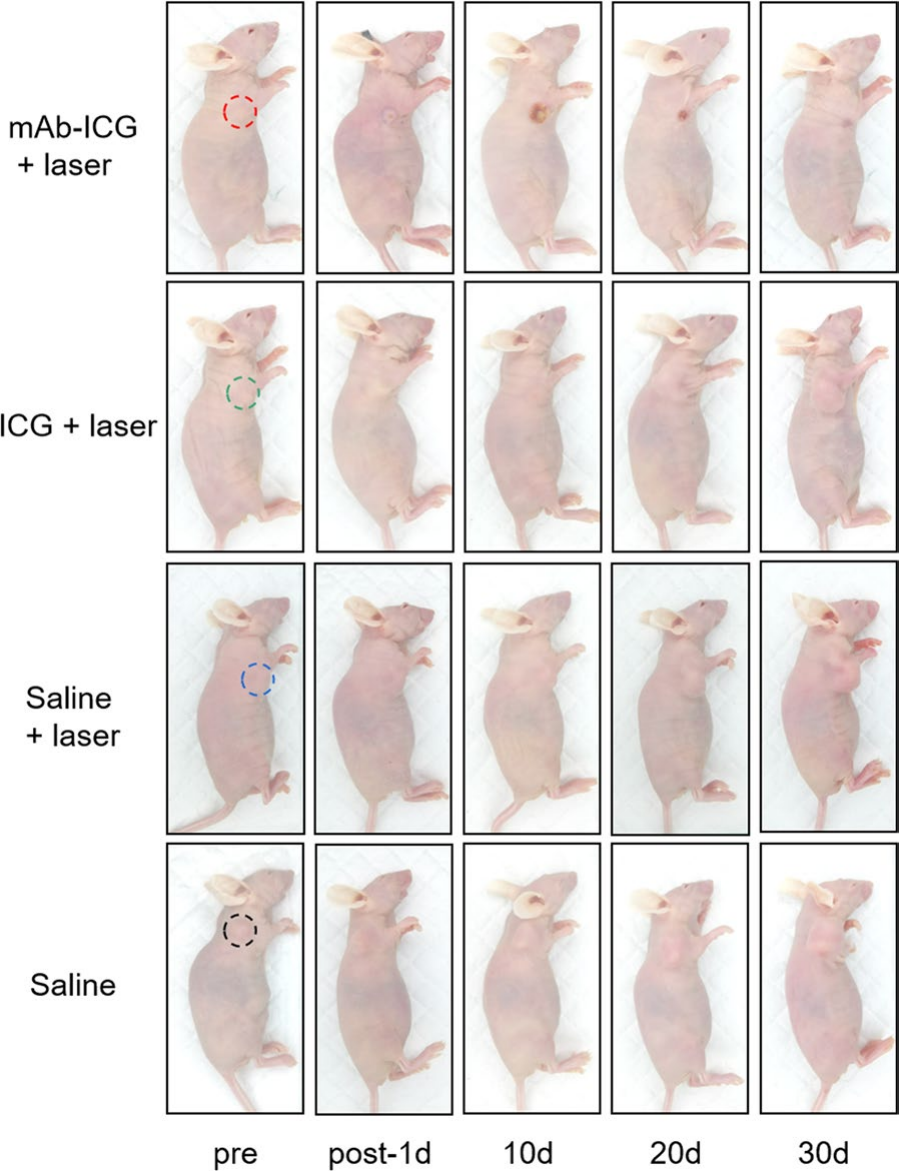



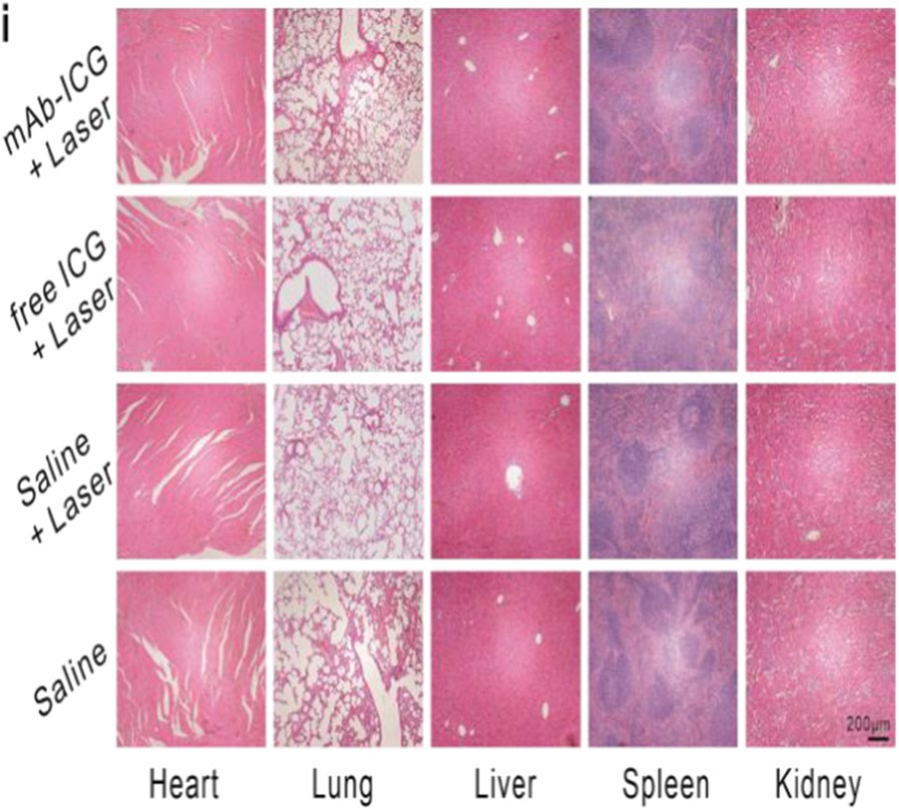


The authors apologise for this error.

The original article [1] has been corrected.

## References

[1] Shao F, Pan Z, Long Y, Zhu Z, Wang K, Ji H, Zhu K, Song W, Song Y, Song X, Gai Y, Liu Q, Qin C, Jiang D, Zhu J, Lan X (2022). Nectin-4-targeted immunoSPECT/CT imaging and photothermal therapy of triple-negative breast cancer. J Nanobiotechnol.

