# Supplementary material for: Nectin-4-targeted immunoSPECT/CT imaging and photothermal therapy of triple-negative breast cancer
Source: J Nanobiotechnology. 2022 May 25;20:243. doi: 10.1186/s12951-022-01444-3 (PMC9131648; doi:10.1186/s12951-022-01444-3)
Supplement: Supplementary file 1 — Additional file 1: Figure S1. The radiolabelling yield (a prior PD-10 purification) and radiochemical purity (b post PD-10 purification) of 99mTc-HYNIC-mAbNectin-4. Figure S2. The tumor-muscle ratio of radioactivity in xenograft tumor-bearing mice on microSPECT/CT images at different time points, n = 3. Figure S3. The viabilities of MCF-7 cells after different treatments, n = 5. Figure S4. The fluorescence quantification of the xenograft tumor ROIs (a) and the tumor-muscle ratio of FL intensity (b) in xenograft-bearing mice on in vivo FL images at different time points, n = 3, ***p < 0.001, ****p < 0.0001. Figure S5. The temperature–time curves of xenograft tumor sites during different treatment. [file 12951_2022_1444_MOESM1_ESM.docx]

**Additional file 1**


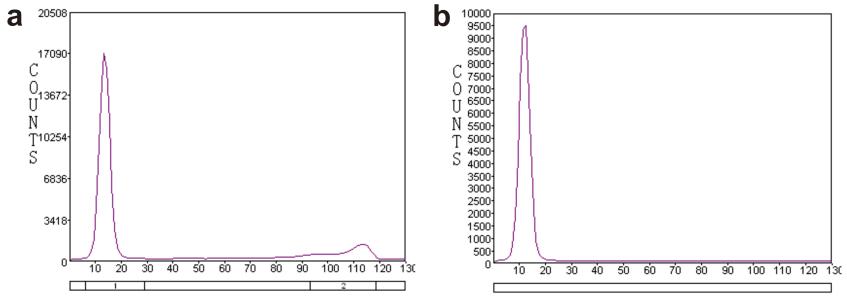

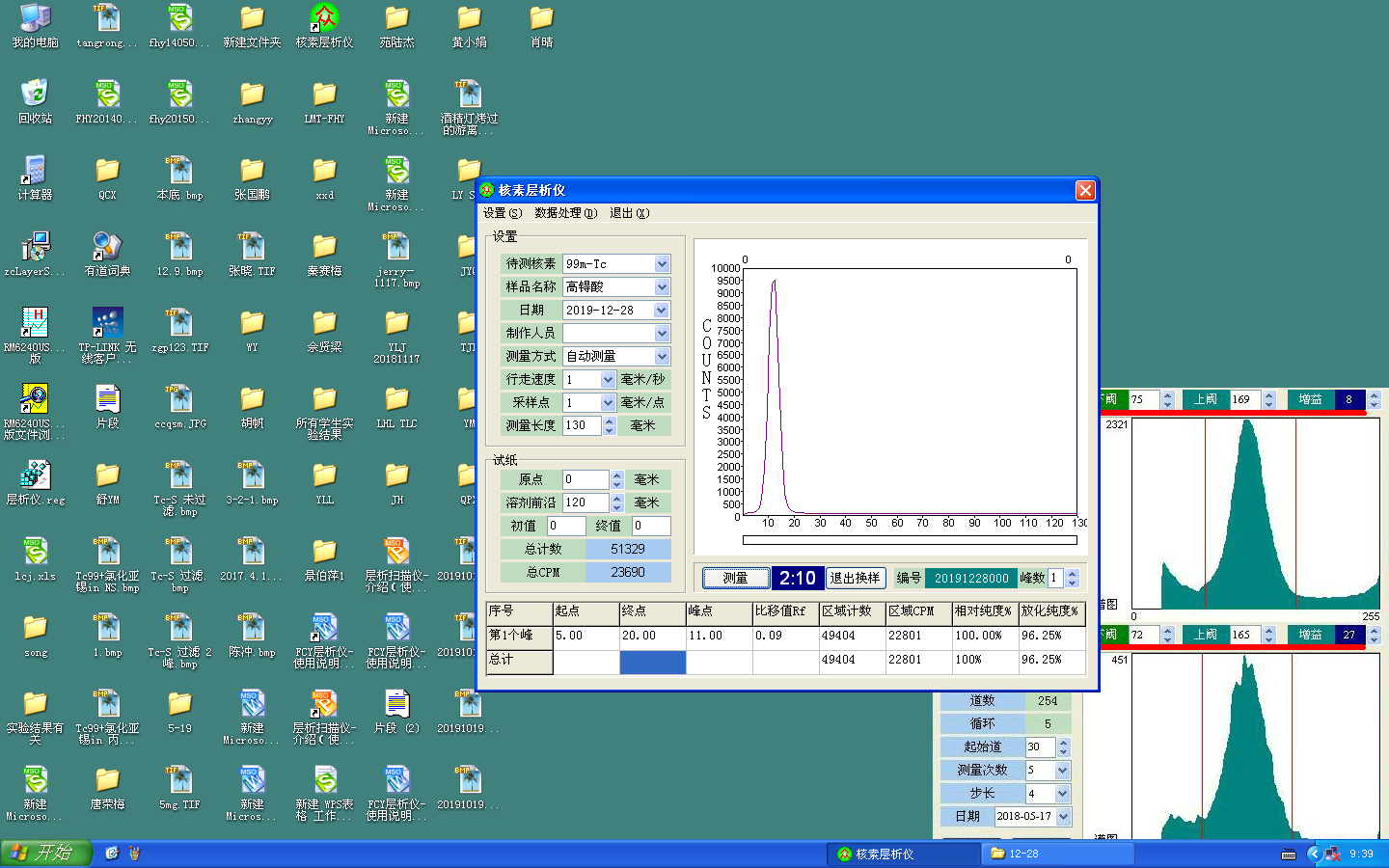


**Figure S1.** The radiolabelling yield (a, prior PD-10 purification) and radiochemical purity (b, post PD-10 purification) of ^99m^Tc-HYNIC-mAb_Nectin-4._


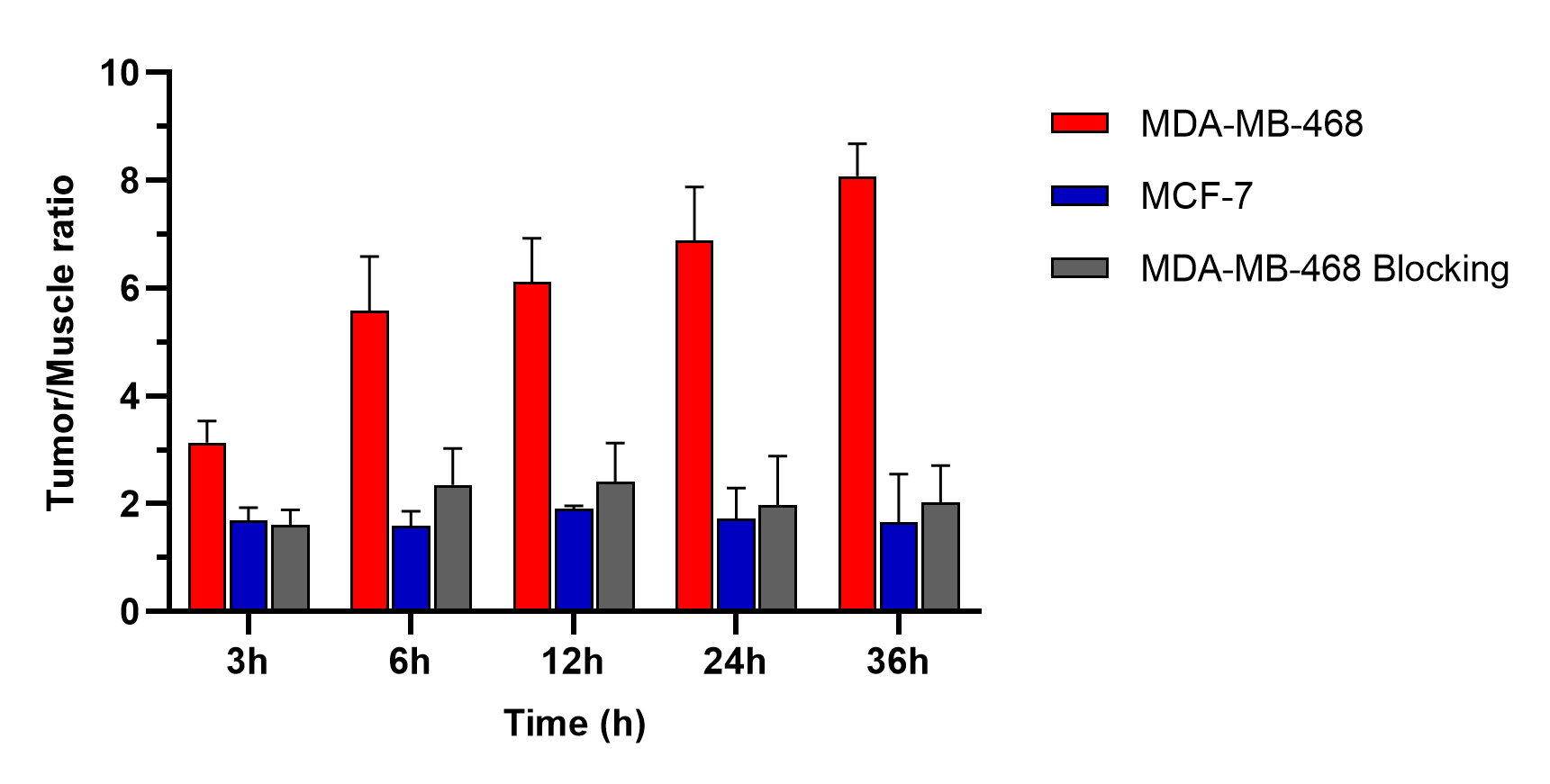


**Figure S2.** The tumor-muscle ratio of radioactivity in xenograft tumor-bearing mice on microSPECT/CT images at different time points, n=3.


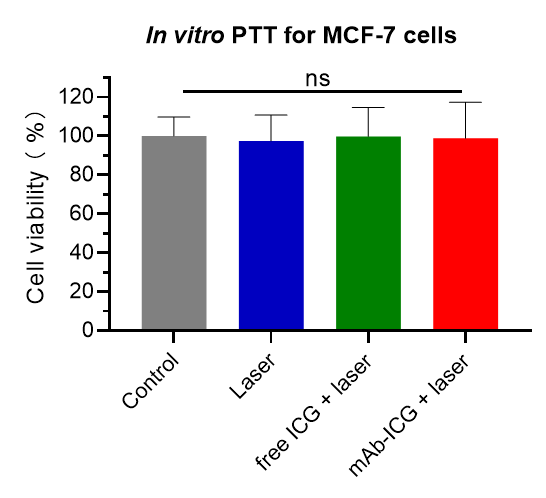


**Figure S3.** The viabilities of MCF-7 cells after different treatments, n=5.


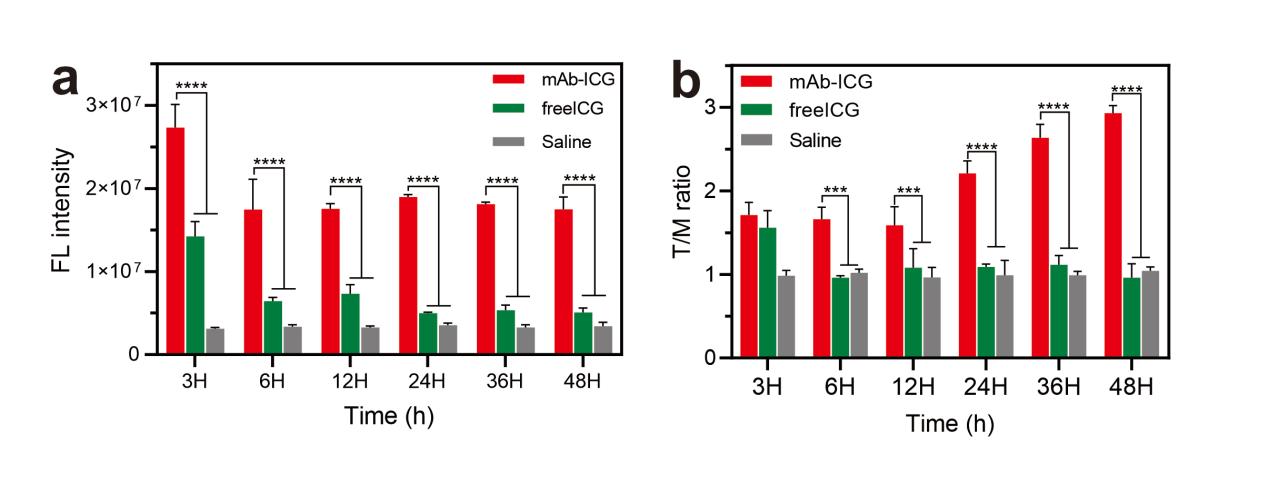


**Figure S4.** The fluorescence quantification of the xenograft tumor ROIs (a) and the tumor-muscle ratio of FL intensity (b) in xenograft-bearing mice on in vivo FL images at different time points, n=3, ****p*＜0.001, *****p*＜0.0001.


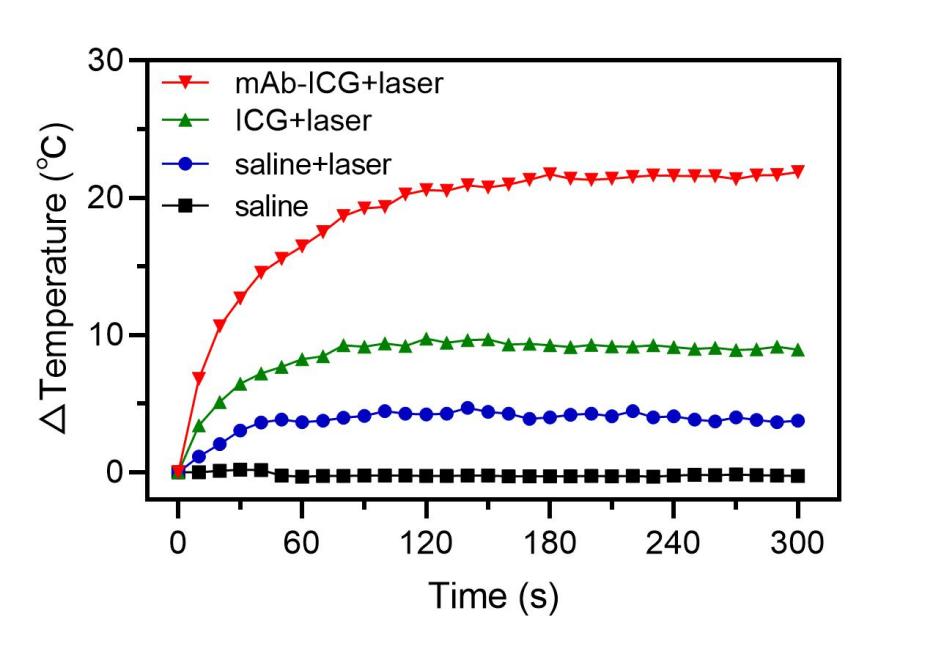


**Figure S5.** The temperature-time curves of xenograft tumor sites during different treatment.
